# Distinctive protein expression in elderly livers in a Sprague–Dawley rat model of normothermic ex vivo liver machine perfusion

**DOI:** 10.1186/s40001-024-01961-x

**Published:** 2024-07-11

**Authors:** Maximilian Zimmer, Karl Herbert Hillebrandt, Nathalie Nora Roschke, Steffen Lippert, Oliver Klein, Grit Nebrich, Joseph Maria George Vernon Gassner, Felix Strobl, Johann Pratschke, Felix Krenzien, Igor Maximilian Sauer, Nathanael Raschzok, Simon Moosburner

**Affiliations:** 1 Department of Surgery, Experimental Surgery, Charité–Universitätsmedizin Berlin, Corporate Member of Freie Universität Berlin and Humboldt-Universität Zu Berlin, Berlin, Germany; 2 BIH Academy, Clinician Scientist Program, Berlin Institute of Health at Charité–Universitätsmedizin Berlin, Berlin, Germany; 3 Center for Regenerative Therapies, Core Unit Imaging Mass Spectrometry, Berlin Institute of Health at Charité, Berlin, Germany

**Keywords:** Liver transplantation, Proteomics, Oxidative stress, Normothermic machine perfusion

## Abstract

**Background:**

Liver grafts are frequently declined due to high donor age or age mismatch with the recipient. To improve the outcome of marginal grafts, we aimed to characterize the performance of elderly vs. young liver grafts in a standardized rat model of normothermic ex vivo liver machine perfusion (NMP).

**Methods:**

Livers from Sprague–Dawley rats aged 3 or 12 months were procured and perfused for 6 h using a rat NMP system or collected as a reference group (*n* = 6/group). Tissue, bile, and perfusate samples were used for biochemical, and proteomic analyses.

**Results:**

All livers cleared lactate during perfusion and continued to produce bile after 6 h of perfusion (614 mg/h). Peak urea levels in 12-month-old animals were higher than in younger animals. Arterial and portal venous pressure, bile production and pH did not differ between groups. Proteomic analysis identified a total of 1477 proteins with oxidoreductase and catalytic activity dominating the gene ontology analysis. Proteins such as aldehyde dehydrogenase 1A1 and 2-Hydroxyacid oxidase 2 were significantly more present in livers of older age.

**Conclusions:**

Young and elderly liver grafts exhibited similar viability during NMP, though proteomic analyses indicated that older grafts are less resilient to oxidative stress. Our study is limited by the elderly animal age, which corresponds to mature but not elderly human age typically seen in marginal human livers. Nevertheless, reducing oxidative stress could be a promising therapeutic target in the future.

**Supplementary Information:**

The online version contains supplementary material available at 10.1186/s40001-024-01961-x.

## Background

Liver transplantation is the sole curative treatment for end-stage liver disease and its demand remains high. Globally, the frequency of liver transplants rose from 23,986 in 2012 to 37,436 in 2022. However, at the end of 2022, more than 11,000 patients were still listed for liver transplantation in the United States and Europe alone [1–3]. Despite the pressing need, a significant number of liver grafts are deemed unsuitable for transplantation due to issues like advanced donor age, hepatic steatosis, or high liver enzyme levels [4–6]. This supply–demand imbalance contributes to elevated mortality rates on the waitlist. One approach to mitigate this gap is increasing the use of livers from extended criteria donors (ECD). Yet, grafts from ECDs are linked to a heightened risk of primary non-function and early allograft dysfunction [7–9]. To assess these high-risk grafts, the adoption of hypothermic and normothermic ex vivo liver machine perfusion (NMP) is gaining ground in clinical settings. While the advantages of perfusion in liver transplantation are being discussed, research continues on determining the optimal perfusion approach, encompassing strategies, such as back-to-base perfusion post initial cold storage or immediate perfusion, and the best duration and temperature for perfusion [10–13].

In Germany, where organ donations rates are low, up to 75% of liver transplantations are from ECDs, with a significant portion originating from older donors [14]. With increasing life expectancy, the proportion of elderly donors is expected to rise [6]. The definition of “older donor age” remains unclear. While Eurotransplant suggests a threshold of 65 years, this age cutoff does not consistently correlate with higher risks of graft dysfunction or complications [15, 16]. Although older donor age is associated with increased risks of graft dysfunction, vascular complications, and retransplantation necessity, recent evidence suggests that transplants using older grafts can yield outcomes comparable to those with younger grafts [17]. Successful liver transplantations have been reported for donors as old as 90 years [18, 19]. This highlights the variable nature of age as a donor selection criterion, suggesting that chronological age might not always reflect biological age and organ functionality. Therefore, it is crucial to identify more accurate parameters that better represent biological age and functionality.

This study aimed to examine the liver tissue and bile proteome of young and elderly liver grafts using an NMP system. Owing to the scarcity of human livers for research, we utilized a pre-established rat NMP system for this analysis [20, 21]. This study not only explores the viability of perfusing elderly livers in a smaller animal model but also investigates the effects of NMP on these liver grafts.

## Methods

### Animals and groups

**Fig. 1:**
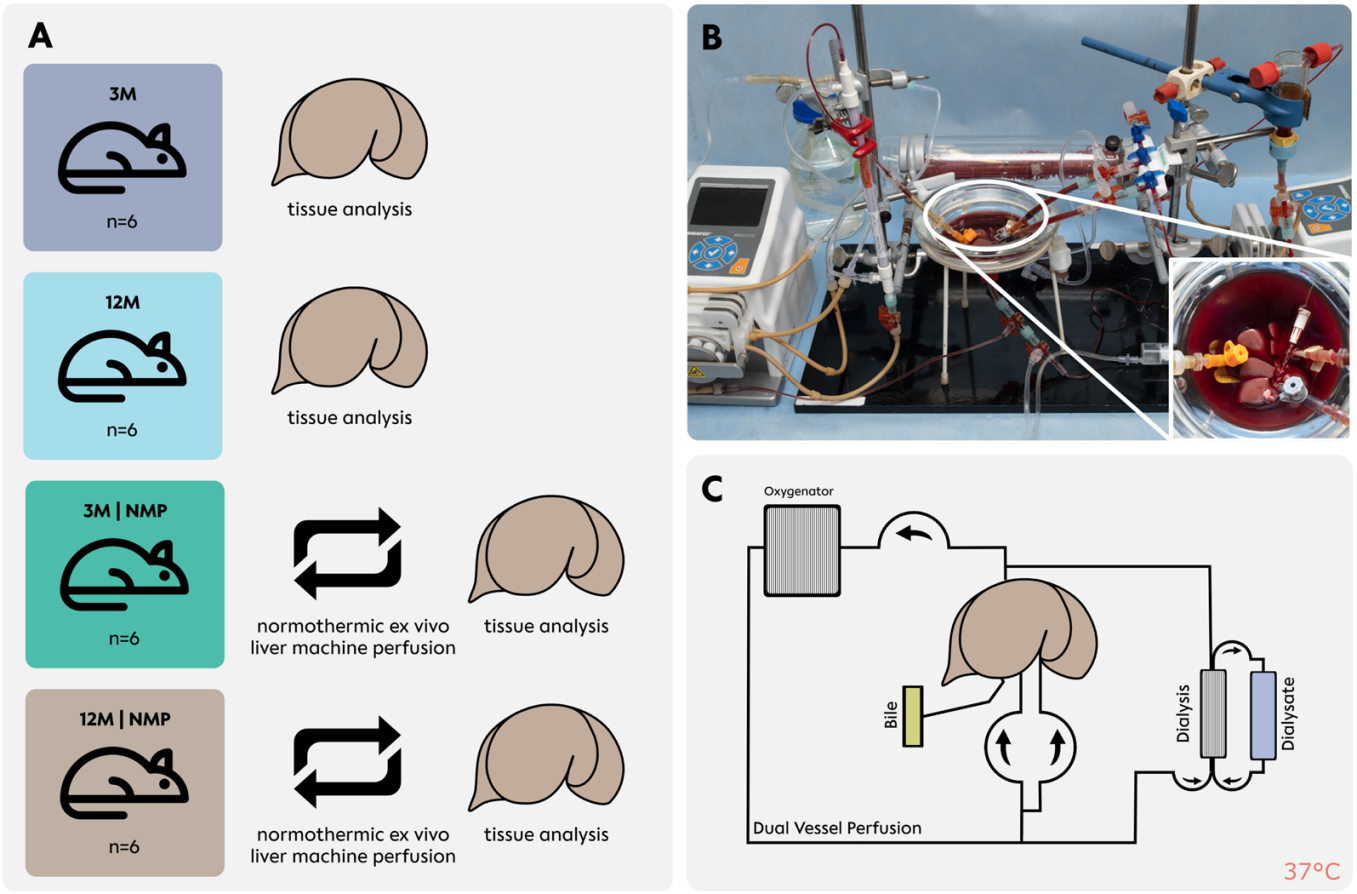
Experimental groups and perfusion set-up. **A** Groups are represented from top to bottom; 3-month-old livers and static cold storage (SCS), 12-month-old livers and SCS, 3-month-old livers and normothermic ex vivo liver machine perfusion (NMP), and 12-month-old livers and NMP**. B** Picture of perfusion set-up with detail shot of liver position and cannulation. Grey—portal vein, orange—inferior vena cava, white—common bile duct, clear—hepatic artery. **C** Schematic of perfusion circuit; perfusion was performed at normothermic (37 °C) temperatures

Male Sprague–Dawley rats were purchased from Janvier (Le Genest-Saint-Isle, France). To ensure adequate acclimatization, experiments were performed after a minimum of 1 week. Animals were kept on a 12-h light–dark circle. 24 rats were randomly assigned to 4 groups (*n* = 6) (Fig. 1). As a reference group, livers from 3- to 12-month-old rats were explanted without following perfusion. Two other groups of 3- and 12-month-old rats were explanted and perfused for 6 h. All procedures were approved by the local authorities for animal welfare and testing (*Landesamt für Gesundheit und Soziales Berlin*, T301/17, G0218/20).

### Liver procurement and perfusion procedure

We used a modified version of a small animal NMP system previously published by our group [20]. In short, the system included a custom-designed perfusion chamber with two circuits for blood oxygenation and dialysis, flow-controlled pumps for dual vessel perfusion (arterial and portovenous) and a silicon membrane oxygenator (*Radnoti LLC., Dublin, Ireland*). The perfusion medium consisted of modified Dulbecco’s Modified Eagle Medium supplemented with several components (Supplementary Table 1), including rat erythrocytes and plasma, and Ci–Ca® dialysate (*Fresenius Medical Care, Bad Homburg, Germany*) was used for the dialysis circuit. Livers were procured under deep anesthesia with inhaled isoflurane and subcutaneous application of buprenorphine and ketamine (Supplementary Material) and stored in a 4 °C Histidine–Tryptophan–Ketoglutarate (HTK) solution until reperfusion in the NMP system [20–22].

### Measurement of biochemical markers and blood gas analysis

Perfusion samples were obtained at the start of the perfusion process, as well as after 3 and 6 h. Arterial blood, venous blood, and dialysate samples were collected. Samples were treated as previously described [21]. Blood gas analysis were measured every 3 h at a point of care device (*ABL800 FLEX, Radiometer GmbH, Berlin, Germany*) and perfusate samples were biochemically analyzed by *Labor Berlin–Charité Vivantes GmbH* for alanine–aminotransferase (ALT), aspartate–aminotransferase (AST), lactate dehydrogenase (LDH), and bilirubin.

### Bile collection and analysis

Bile was continuously collected during perfusion, weight and bile pH were measured hourly using the PCE-228 pH-Meter (PCE Deutschland GmbH, Meschede, Deutschland). After perfusion the bile was stored at − 80 °C. Using a LDH Activity Assay Kit (*Sigma-Aldrich, St. Louis, USA*), we quantified LDH activity in the bile for the samples collected 1, 3 and 6 h after start of perfusion following the manufacturer’s instructions. Duplicates were measured on an *Infinite 200 PRO* plate reader (*Tecan Group, Männedorf, Switzerland*).

### Tissue sampling, triglyceride histological analysis

After the perfusion one sample of each lobe was fixed in formaldehyde and a minimum of two samples of each lobe were freshly frozen for analysis. For histological analysis we choose the following staining methods: Hematoxylin–Eosin (H.E.), Sirius Red (both *AppliChem, Darmstadt, Germany*), TdT-mediated dUTP-biotin nick end labeling (TUNEL, *Hoffmann-La Roche AG, Basel, Switzerland*), and Sudan red (*Abcam, Cambridge, UK*). Staining was performed on 3 µm thick formaldehyde fixed slides except for Sudan, which was made with 4 µm thick freshly frozen slides to ensure adequate lipid representation. Liver vitality, loss of nucleus, and cellular fragmentation as well as portal fields for sinusoidal dilatation were assessed. Sirius Red Staining was performed for semiquantitative assessment of collagen. TUNEL and counterstaining with 4ʹ,6-Diamidino-2-phenylindol (DAPI, *Thermo Fisher Scientific Inc., Waltham, Massachusetts, USA*) was performed for assessment of tissue damage. Staining was performed according to the manufacturer’s instructions.

Semi-quantitative assessment of Sirius red staining was performed with Fiji software (Fiji Is Just ImageJ, ImageJ2) utilizing a color threshold method [23]. Snap frozen tissue sections were used for triglyceride determination with a standardized kit (free glycerol reagent and triglyceride reagent both from *Sigma-Aldrich, St. Louis, USA*). We quantified total triglyceride content in the liver tissue after mechanical and thermal homogenization, centrifugation and 1:10 dilution and following the manufacturer’s instructions. Duplicates were measured on an *Infinite 200 PRO* plate reader at 540 nm (*Tecan Group, Männedorf, Switzerland*.)

### Proteomic analysis and western blot verification

Approximately 5 mg of rat liver per tissue issue per experiment was homogenized lyophilized and stored at − 80 °C. Bile aliquots after 6 h of perfusion were prepared for analysis as described by Ciordia et al. [24]. For both sample types, protein extraction, digest, and peptide desalting were performed by filter-aided sample preparation (FASP) method [25]. For protein identification 2 µL eluate was injected into a *NanoHPLC* (*Dionex UltiMate 3000, Thermo Fisher Scientific, Waltham, MA, USA*) coupled to an ESI–QTOF ultra high-resolution mass spectrometer (*Impact II, Bruker Daltonic GmbH, 28359 Bremen, Germany*). Data were quantified and analyzed using *ProteinScape* (*Version 3.0, Bruker Daltonic GmbH, 28359 Bremen, Germany*) as descripted before (Supplementary Material) [26]. Gene ontology (GO) enrichment analysis were performed using the rat genome database of the Multi Ontology Enrichment Tool (MOET) version 2 [27]. A minimum of two unique peptides per protein were required. In brief, derived peak lists of the acquired raw files were searched against the Swiss-Prot database of rattus norvegicus. The significance threshold was set *p* < 0.05. A homology threshold type was chosen with a false discovery rate of 1% on both peptide and protein level. Peptide mass tolerance was set to 10 ppm and the fragment ion mass tolerance to 0.05 Da. A minimum of two unique peptides per protein was required.

Exponentially modified protein abundance index (emPAI) was used for estimation of proteomics composition in bile analysis. EmPAI values are normalized by the number of theoretical peptides for each protein, which allows for comparison of different proteins within a single sample [28, 29]. All identified proteins were screened according to their unique UniProt identifier and excluded from further analysis if present in less than half (*n* = 3) of experiments in each group. Proteomic data are available on the ProteomeXchange Consortium via the PRIDE partner repository (Project accession: PXD045394). We performed western blot analysis for aldehyde dehydrogenase 1A1, transferrin, peroxiredoxin, vimentin, 2-Hydroxyacid oxidase 2 and used actin and alpha-tubulin as housekeeping proteins (Supplementary Material).

Anti-alpha-tubulin antibody (T9026, diluted 1:500) (both *Sigma-Aldrich, St. Louis, USA*).

### Statistical analysis

Statistical analysis was performed using *R* (version 4.1.2) and *R* Studio (version 2021.09.0) for macOS (R Foundation for Statistical Computing, Vienna, Austria) [30]. Additional required packages for graph plotting and analysis were tidyverse and gtsummary. Graphs with time-dependent data show pairwise comparisons at each analysis timepoint (*ns* not significant, *p < 0.05, **p < 0.01) as a violin plot with individual values. Groups were compared using the Mann–Whitney *U* test or the Kruskal–Wallis test. Differentially abundant proteins were identified using ANOVA. Data are reported as median and interquartile range (IQR). A *p* < 0.05 was considered statistically significant.

## Results

### Animal characteristics and perfusion parameters

Elderly rats as well as their livers were significantly heavier than their younger counterparts (animal weight: 530 g vs. 779 g, *p* < 0.001; liver weight: 16.9 g vs. 20.7 g, *p* < 0.001). After recovery all livers had a maximum WIT of 5 min. The cold ischemia time (CIT) of all 24 livers was below 1 h. In both perfusion groups arterial and portal venous pressure were in physiological range and did not differ between groups. Post perfusion flush macroscopy was satisfactory, except one common spot of storage damage at the right medial lobe.

### Perfusate analysis

**Fig. 2:**
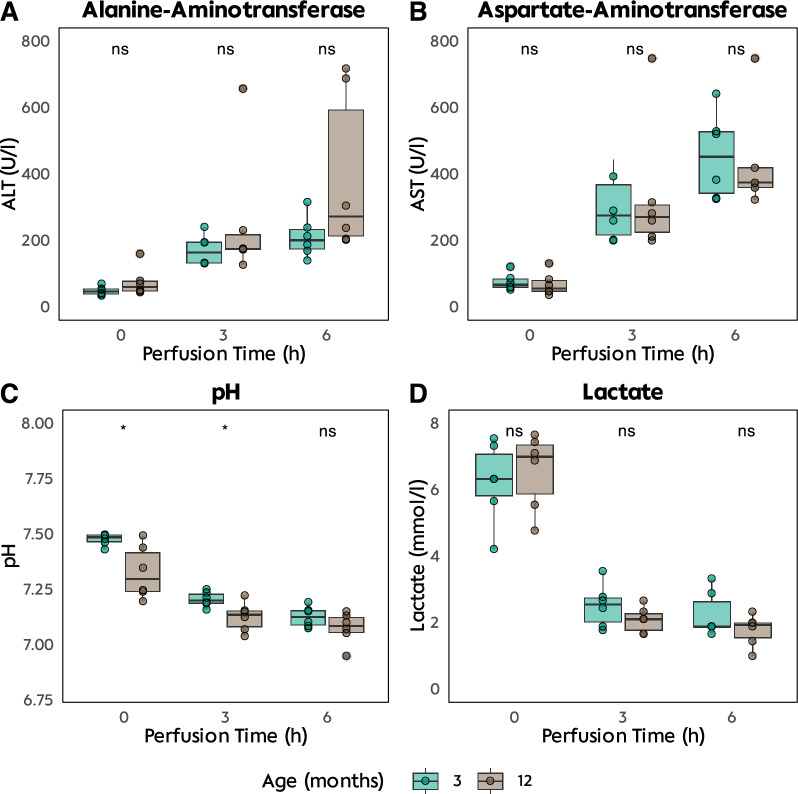
Perfusate gathered normothermic perfusion: Analysis for 6 h perfusion of elderly (12-month-old) and young (3-month-old) Sprague–Dawley rats. **A** Aspartate–aminotransferase levels (AST) **B** Alanine–aminotransferase levels (ALT) **C** pH levels **D** Lactate clearance

Liver tissue damage was measured by ALT and AST did not differ between groups and were highest after 6 h of perfusion with peak ALT at 226 U/l (3M|NMP vs. 12M|NMP: 272 U/l vs. 200 U/L; *p* = 0.2, Fig. 2). Global cellular damage by LDH was highest after 6 h of perfusion with a mean of 5,278 U/l. Elderly livers had significantly higher urea levels to the end of perfusion (median: 35.5 U/l vs. 45.5 U/l; *p* = 0.036). Perfusion started with an arterial pH of 7.45 and ended up with an overall value of 7.1. Blood gas analysis showed sufficient oxygenation of the perfusate and a functional metabolism (after 6 h: arterial pO2 445 mmHg, venous pO2 38 mmHg). During perfusion, all livers cleared lactate constantly (6.3 mmol/l IQR 2 mmol/l to 1.75 mmol/l IQR 0.37 mmol/l). Elderly livers started with slightly higher lactate levels (6.9 mmol/l vs. 5.8 mmol/l; *p* = 0.6), however both groups cleared up to 1.75 mmol/l after 6 h of perfusion (*p* = 0.4) (Supplementary Table 2).

### Bile production analysis

**Fig. 3:**
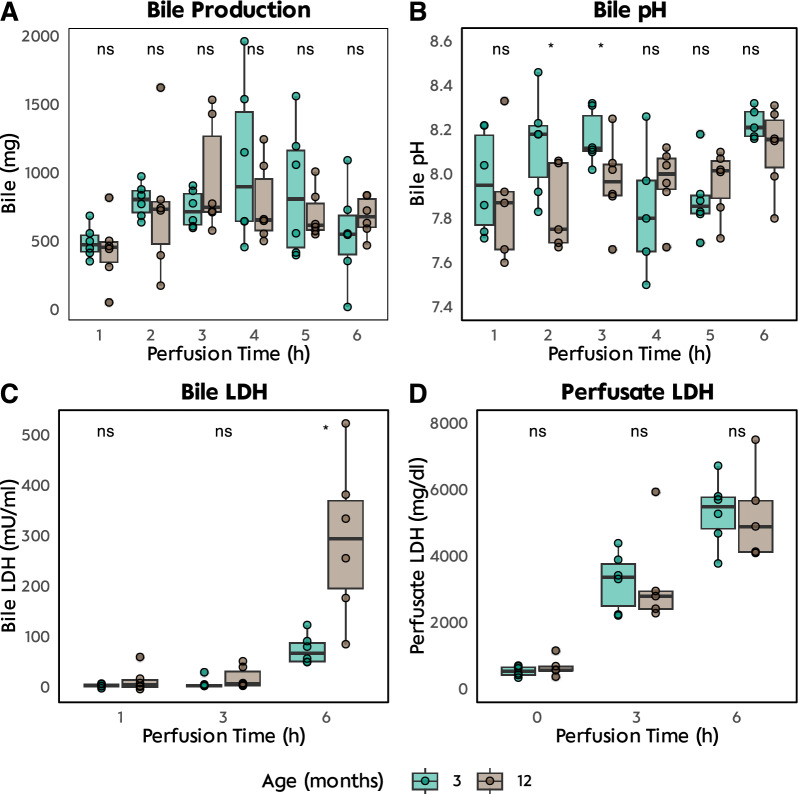
Bile: Analysis of production and cellular damage. **A** Hourly bile production (mg) **B** Hourly bile pH **C** Levels of bile lactate-dehydrogenase (LDH) **D** Levels of LDH in the perfusate

Bile production ranged from 447 mg/h (IQR 158 mg) at the start of perfusion to 758 mg/h after 2 h of perfusion (IQR 236 mg, Fig. 3). Despite livers of elderly rats being of larger size, they did not produce significantly more bile. Overall bile pH ranged between 7.86 and 8.06. Bile LDH levels were significantly higher in elderly liver grafts (295 mU/ml vs. 69 mU/ml, *p* = 0.009) after 6 h of perfusion.

### Bile proteome characterization

**Fig. 4:**
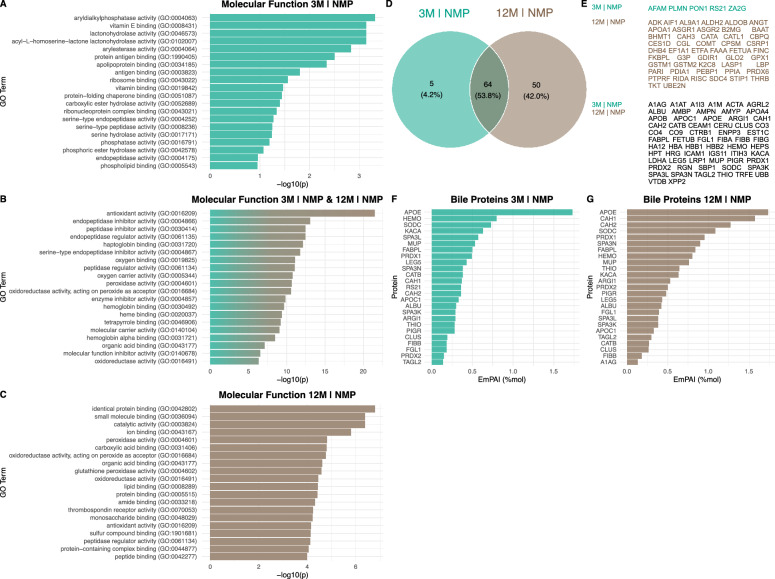
Bile: Gene ontology enrichment analysis of proteomic results **A** cellular compartment **B** molecular function and **C** biological process. **D** Venn diagram for proteins found in proteomic analysis of bile and **E** individual proteins labeled for “D”. **F** Most expressed protein in bile of 3-month-old perfused livers and **G** Most expressed proteins in bile of 12-month-old livers

Proteomic analysis of bile from the NMP-treated groups identified a total of 119 distinct proteins. Of these, a mere five proteins—AFAM, PLN, PON1, RS21, and ZA2G—were exclusively detected in the bile of the young control group. Interestingly, there was a considerable overlap in the bile proteome, with 54% of the proteins being common to both age groups (Fig. 4). However, 50 proteins were uniquely expressed in the bile from the elderly group.

In terms of protein abundance measured by the exponentially modified Protein Abundance Index (emPAI), APOE emerged as the most prevalent protein in both age groups. This was followed by CAH1/CAH2 and SODC in the 12-month-old animals, and HEMO and SODC in the 3-month-old animals. GO analysis revealed that antioxidant activity and endopeptidase inhibitor activity were the most significant biological processes represented in the bile proteome.

### Histological analysis

H&E staining revealed well-preserved liver tissues in all samples, showing distinct lobular structures and portal triads without significant necrosis, though perfused livers displayed minor sinusoidal dilation. Sirius Red staining highlighted the Glisson capsule and minor increases in intraparenchymal collagen in elderly livers, but semi-quantitative analysis showed no significant differences between 3- and 12-month-old livers (1.72% vs. 1.17%; *p* = 0.9). Both age groups exhibited minimal lipid signals, unaffected by perfusion, as supported by consistent triglyceride levels in tissue analysis (Supplementary Fig. 1).

**Fig. 5:**
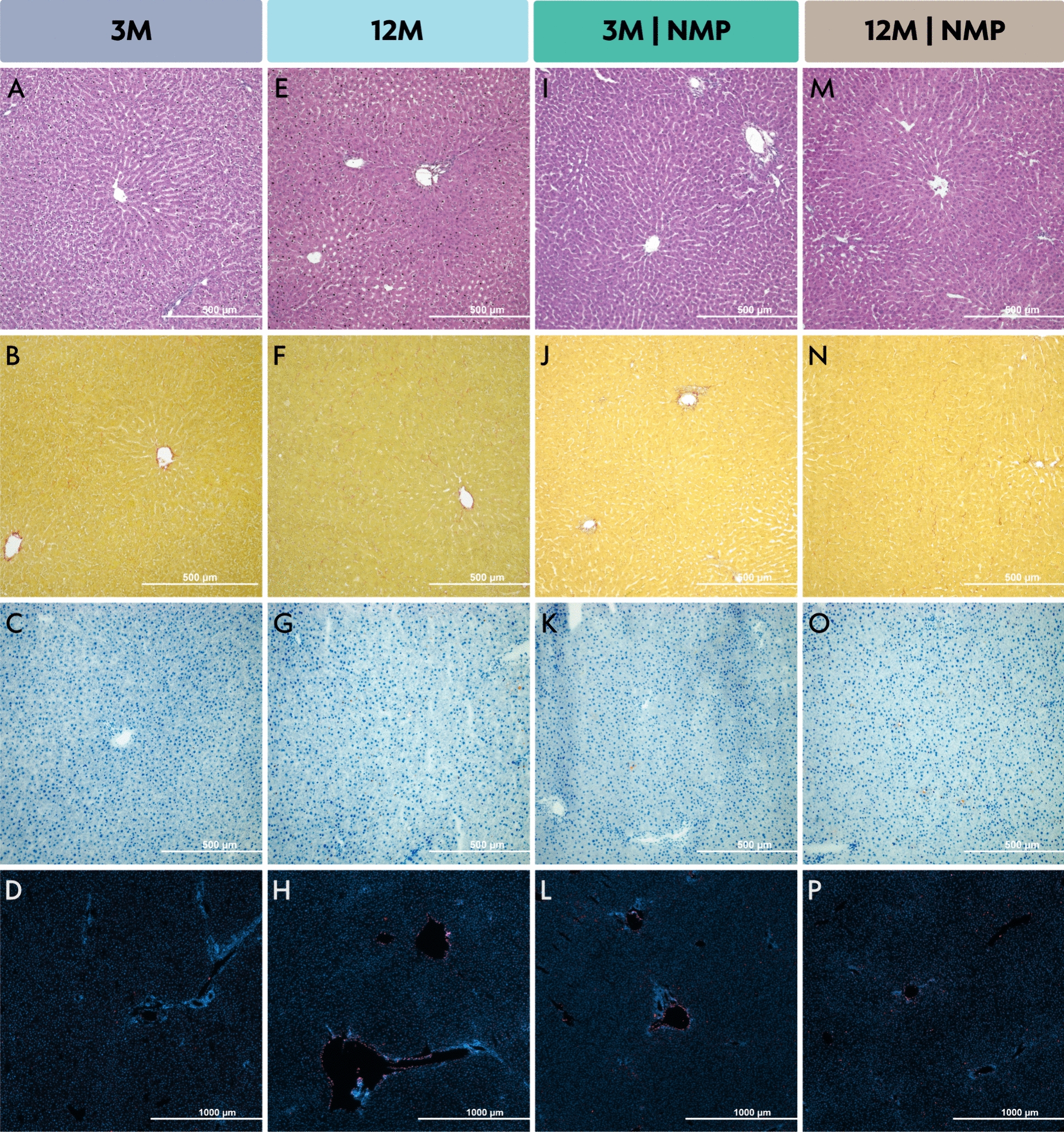
Liver tissue: Histological analysis of experimental groups. Each row represents a different staining. From top to bottom: H&E, Sirius red, Sudan red, TUNEL. Groups are represented from left to right: 3-month-old liver after static cold storage (SCS), 12-month-old liver after SCS, 3-month-old liver after normothermic ex vivo liver machine perfusion (NMP), and a 12-month-old liver after NMP

Elderly livers displayed more apoptosis near the liver capsule compared to younger livers, and perfused livers showed slightly increased apoptosis in both the capsule and parenchyma than the control group. Despite some isolated apoptotic signals, overall distribution was homogeneous with occasional smaller apoptotic areas (Fig. 5).

### Proteomic analysis

**Fig. 6:**
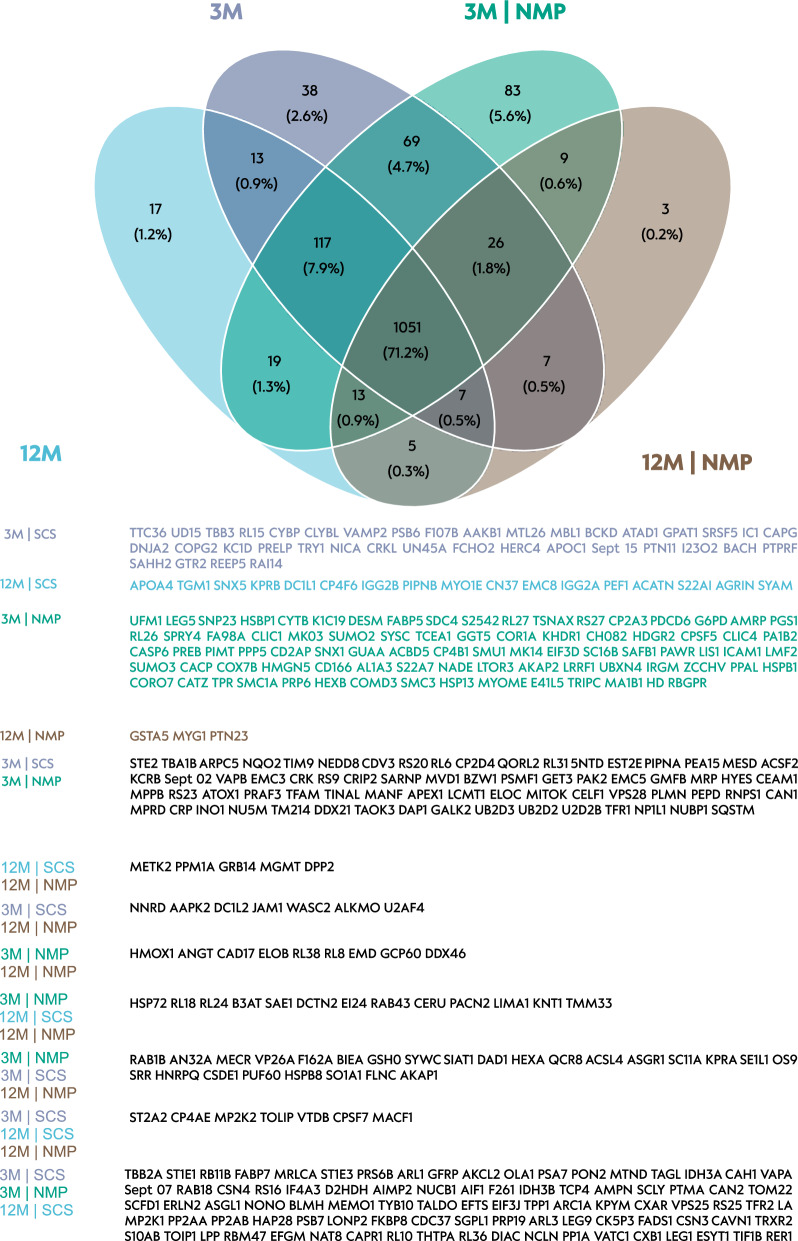
Liver tissue: Venn diagram of label-free quantification proteomic analysis of perfused and non-perfused control livers. Experimental groups were 3-month-old livers (3M) and static cold storage (SCS), 12-month-old livers and SCS, 3-month-old livers and normothermic ex vivo liver machine perfusion (NMP), and 12-month-old livers and NMP. Represented below are the proteomic intersections of each group

We identified 1477 proteins in the analyzed liver tissue (Fig. 6), with the majority (71.2%) being common across all experimental groups. The largest protein overlap was observed among the groups 3M, 3M|NMP, and 12M (7.9%), while the smallest overlap occurred within the elderly liver group (0.3%). Specifically, five proteins (METK2, PPM1A, GRB14, MGMT, DPP2) were unique to elderly livers, and three proteins (GSTA5, MYG1, PTN23) were exclusive to elderly and perfused livers.

GO enrichment analysis indicated that oxidoreductase activity, catalytic activity, and small molecule binding were predominant molecular functions in the proteomic data set. In terms of biological processes, small molecule, oxoacid, and organic acid metabolic processes were most relevant (Supplementary Fig. 2). A targeted approach cross-referenced proteins in pathways of interest. For the inflammatory response, increased levels of fibrinogen alpha chain, haptoglobin, and serine protease inhibitor A3N were noted in livers treated with NMP, with a decrease in Serotransferrin levels (Supplementary Fig. 3). Regarding aging and cellular senescence, elevated levels of Acyl-CoA (8-3)-desaturase were found in elderly livers, and decreased levels of Sulfotransferase 2A1 were observed, irrespective of NMP treatment (Supplementary Fig. 4). Aldehyde dehydrogenase 1A1, a marker of cellular detoxification, was heightened in elderly livers (Supplementary Fig. 5). These proteomic results were corroborated with western blot analysis for three representative proteins (Supplementary Fig. 6): aldehyde dehydrogenase 1A1 was consistently elevated in elderly livers, Vimentin was marginally present in young livers, and Serotransferrin was more abundant in livers post-NMP, regardless of the age of the animals.

**Fig. 7:**
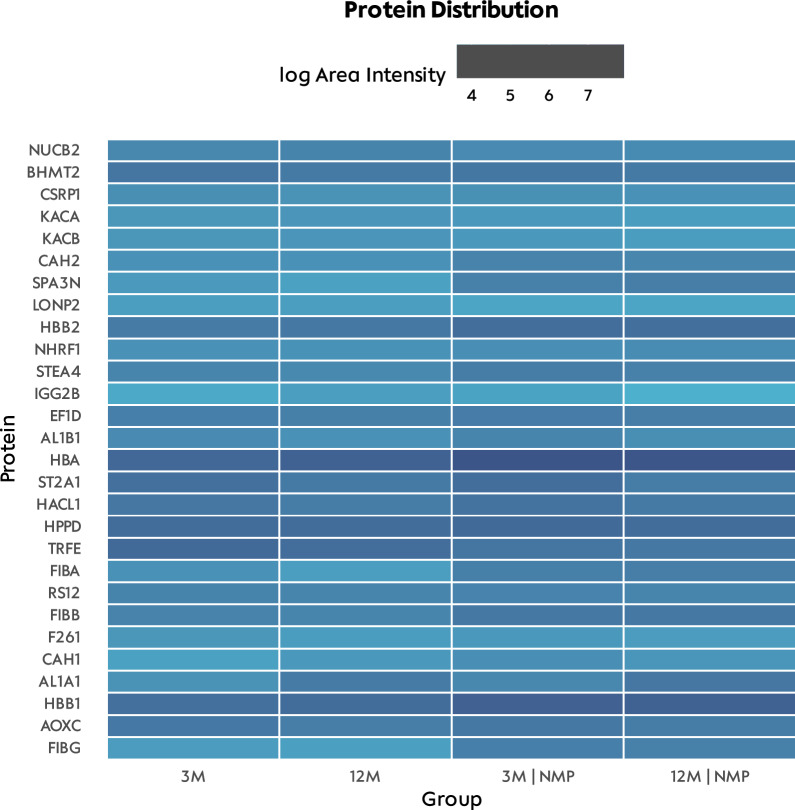
Liver tissue: Heatmap of proteins with the largest intergroup differences. *3M* 3-month-old livers, *SCS* static cold storage, *NMP* normothermic ex vivo liver machine perfusion

**Fig. 8:**
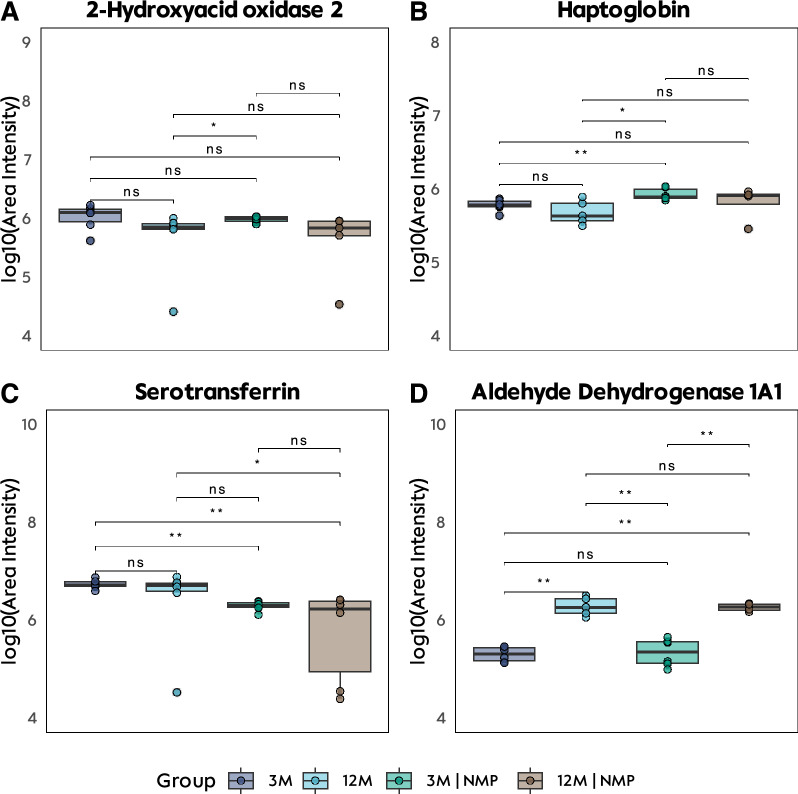
Liver tissue: Exemplary proteins analyzed after gene ontology enrichment analysis and research of literature. *3M* 3-month-old livers, *SCS* static cold storage, *NMP* normothermic ex vivo liver machine perfusion

Heatmap analysis of proteins with the most significant intergroup differences revealed that hemoglobin, fibrinogen, carbonic anhydrase 1, and aldehyde dehydrogenase 1A1 were notably prominent in both perfused groups (Fig. 7). After an extensive review of literature and our GO analysis and heatmap results, four proteins were selected for downstream analyses: 2-Hydroxyacid oxidase 2, haptoglobin, Serotransferrin, and aldehyde dehydrogenase 1A1 (Fig. 8). Age-related effects were evident for 2-Hydroxyacid oxidase 2, vimentin, and aldehyde dehydrogenase 1A1, while NMP-associated differences were observed in haptoglobin, serotransferrin, and peroxiredoxin-2.

## Discussion

Liver grafts are often rejected due to either the advanced age of the donor or a significant age difference between the donor and the recipient. This adds to the persistent challenge of the shortage of appropriate allografts. The objective of this study was to analyze and define the performance parameters of young and elderly liver grafts, based on bile and tissue proteome, using a rat NMP system. A total of 24 livers from Sprague–Dawley rats aged 3 months and 12 months were examined to achieve this goal. Traditional performance parameters, such as perfusion pressure, bile production, and lactate clearance, were equally well-maintained in both elderly and young liver grafts.

However, peak levels of urea in the perfusate were found to be higher in elderly liver grafts, consistent with findings of clinical studies on the perfusion of ECD grafts [9, 19, 31]. Although urea level were slightly elevated, they remained within the reference range, indicating that they did not impact graft quality or allow for graft assessment on their own. Transaminase levels were elevated in both groups, although overall still lower than previously published by our own workgroup, possibly attributed to greater experience in rat NMP [20]. Furthermore, the difference between groups based on ALT values alone would not allow for an adequate differentiation between a viable graft for transplantation or not.

Quality assessment factors during NMP established in clinical trials include bile production and lactate clearance, both of which were present in the NMP groups [32, 33]. In combination with H&E and TUNEL staining, all 12 perfused rat livers would seem viable for transplantation.

After 6 h of NMP, bile LDH levels, which serve as an indicator of cellular damage, were found to be significantly higher in the elderly liver grafts (Fig. 3C) [34]. The biliary tract is particularly vulnerable to ischemia reperfusion injury, which led our own group to adopt a dual vessel perfusion system (hepatic artery and portal vein) to better reflect the clinical standard and provide better protection against bile duct damage. Furthermore, research has demonstrated a correlation between donor age and ischemic-type biliary lesions in humans. This finding, in turn, raises the risk of requiring retransplantation or experiencing long-term complications [35, 36].

We could not identify any histological tissue alterations in elderly liver grafts. No relevant increase in steatosis hepatis or fibrosis were detectable in the Sudan and Sirius red staining. Induction of fibrosis and steatosis hepatis in rats usually requires pharmacological treatment or changes in diet [37, 38]. Age alone does not cause liver fibrosis and steatosis alone and therefore reflects the clinical dilemma of variability of age and its correlation with organ function.

To conduct a more comprehensive analysis of elderly liver grafts, we performed proteomic analysis on liver tissue after NMP and compared it to tissue samples from non-perfused controls. Our findings revealed that proteins associated with cellular detoxification and oxidoreductase activity were more abundant in elderly liver grafts.

Proteins associated with inflammation and metabolic activity were more abundant in grafts treated with NMP. In addition, we found that fibrinogen levels were higher in NMP-treated grafts, regardless of age. Fibrinogen plays a multifaceted role in tissue injury and inflammation, and is involved in several pathways, including blood coagulation [39]. The fact, that perfused livers had higher levels of fibrinogen is most likely attributable to the prolonged mechanical insult and coagulation activation during perfusion. The same was true for serine protease inhibitor A3N which was equally elevated in NMP groups and haptoglobin [40]. Serotransferrin was lower after NMP. Serotransferrin is an acute phase protein relevant for iron transport and has been shown to be upregulated in cases of acute rejection after liver transplantation [41].

Vimentin has been shown to be associated with hepatocellular apoptosis when elevated but also impairing hepatic stellate cell motility and growth, when reduced [42, 43]. Vimentin is an intermediate filament involved in modulating cell morphology, growth, and motility. The higher levels of vimentin observed in the younger livers may indicate greater regenerative capacity.

Finally, aldehyde dehydrogenase 1A1 has been shown to be down-regulated in cases of ischemia reperfusion injury and up-regulated as a defense against oxidative damage induced by reactive lipid aldehydes [44, 45]. In our experiments, we observed significantly elevated levels of aldehyde dehydrogenase 1A1 in 12-month-old livers. This suggest that these livers may require increased protection against oxidative damage. These findings were validated through western blot analysis, which revealed an equal distribution of aldehyde dehydrogenase 1A1.

The analysis of bile collected during NMP showed that APOE and CAH1 were the most abundant proteins. APOE and CAH1 are known regulators of lipid transport and interact with bile acid [46, 47]. In addition, we found that superoxide dismutase (SODC) was present in both groups. Superoxide dismutase is an important antioxidant defense mechanism but can cause excess cellular damage if not regulated. Furthermore, the bile collected from the 12-month-old perfused livers contained 50 additional proteins compared to the control group. One explanation could be the excess cell damage caused in older grafts, activating further downstream pathways, and causing and upregulated protein expression. However, there is currently a lack of available bile proteomic analyses during small animal liver machine perfusion, indicating a need for further exploration and investigation.

Our study is limited using rat liver grafts aged only up to 12 months. As such, the applicability of our findings to clinically relevant donor ages, particularly those over 70 years, is limited. In addition, the extended husbandry times required for animals to reach even older ages, combined with a higher incidence of neoplastic occurrences, limited our ability to analyze animals of even more advanced age. Although there are other rat strains that are more suitable for aging research, we chose the rat strain based on the comparability with the NMP system and rat liver transplantation setup, and the results of perfusate analysis. Nevertheless, our study demonstrates changes that are already apparent on the proteomic level and during perfusion after 12 months of aging.

## Conclusions

Liver grafts from 3- to 12-month-old Sprague–Dawley rats showed comparable performance on NMP. However, proteomic analysis of liver tissue and bile samples revealed differences that suggest older age impairs the ability to cope with oxidative stress during liver machine perfusion. To gain further insights, future studies should investigate the outcomes after transplantation of old vs. young rat livers using static cold storage vs. NMP. Moreover, these studies should focus on reducing oxidative stress as a therapeutic target.

### Supplementary Information

Supplementary Material 1.

## Data Availability

The data sets used and/or analyzed during the current study are available from the corresponding author on reasonable request or are provided in the supplementary material. Proteomic data is available on ProteomeXchange Consortium via the PRIDE partner repository (Project accession: PXD045394).

## Abbreviations

ALT: Alanine-aminotransferase
AST: Aspartate-aminotransferase
CIT: Cold ischemia time
DAPI: 4ʹ,6-Diamidino-2-phenylindol
DMEM: Dulbecco’s modified eagle medium
ECD: Extended criteria donor
emPAI: Exponentially modified protein abundance index
H.E.: Hematoxylin–Eosin
HTK: Histidine–Tryptophan–Ketoglutarate
GO: Gene ontology enrichment
IQR: Interquartile range
LDH: Lactate dehydrogenase
TUNEL: TdT-mediated dUTP–biotin nick end labeling
NMP: Normothermic ex vivo machine perfusion
UNOS: United Network for Organ Sharing
WIT: Warm ischemia time
