# Supplementary material for: Distinctive protein expression in elderly livers in a Sprague–Dawley rat model of normothermic ex vivo liver machine perfusion
Source: Eur J Med Res. 2024 Jul 11;29:361. doi: 10.1186/s40001-024-01961-x (PMC11238374; doi:10.1186/s40001-024-01961-x)
Supplement: Supplementary file 1 — Supplementary Material 1. [file 40001_2024_1961_MOESM1_ESM.pdf]

Supplementary Material

Distinctive Protein Expression in Elderly Livers in a Sprague Dawley Rat Model of Normothermic *ex vivo* Liver Machine Perfusion

Authors: Maximilian Zimmer[1], Karl H. Hillebrandt[1,2], Nathalie N. Roschke[1], Steffen Lippert[1], Oliver Klein[3], Grit Nebrich[3], Joseph MGV Gassner[1,2], Felix Strobl[1], Johann Pratschke[1], Felix Krenzien[1,2], Igor M. Sauer[1], Nathanael Raschzok[1,2], Simon Moosburner[1,2]

Abbreviations:

3M:	3-month-old livers
12M:	12-month-old livers
3M | NMP:	3-month-old livers and normothermic ex vivo liver machine perfusion (NMP)
12M | NMP:	12-month-old livers and NMP

Supplementary Figure 1

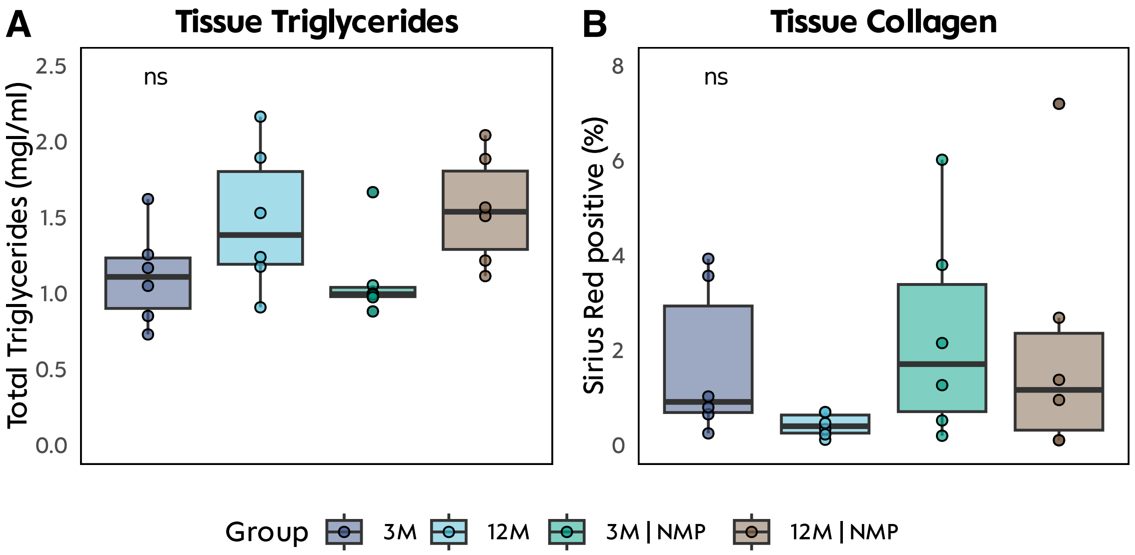

A Triglyceride content in liver tissue after perfusion, which did not differ between age of animals or if livers were perfused or not **B** Tissue collagen levels measured as Sirius Red positive staining percentage.

Supplementary Figure 2

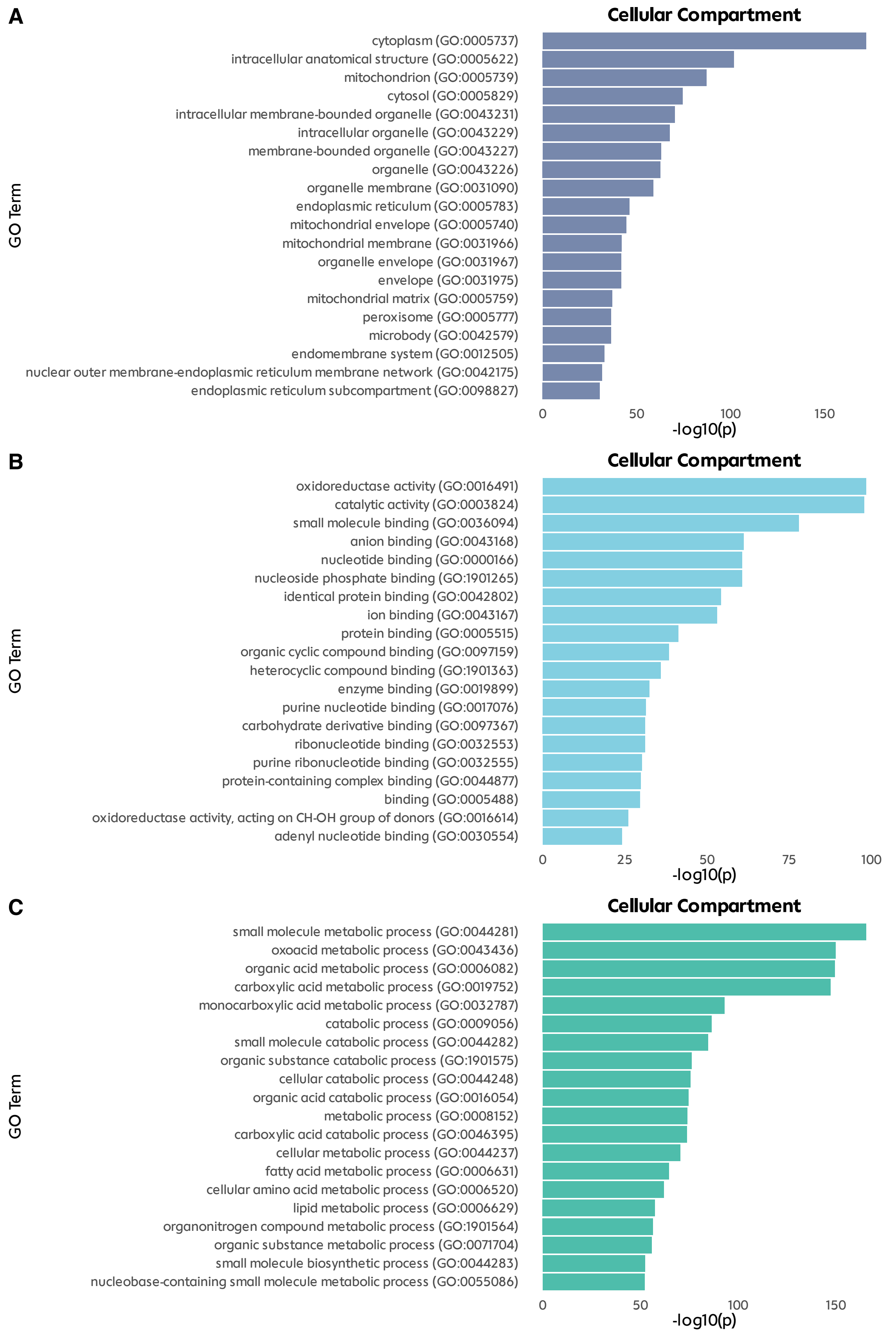

Gene ontology enrichment analysis of proteomic results of liver tissue for **A** cellular compartment **B** molecular function and **C** biological process.

Supplementary Figure 3

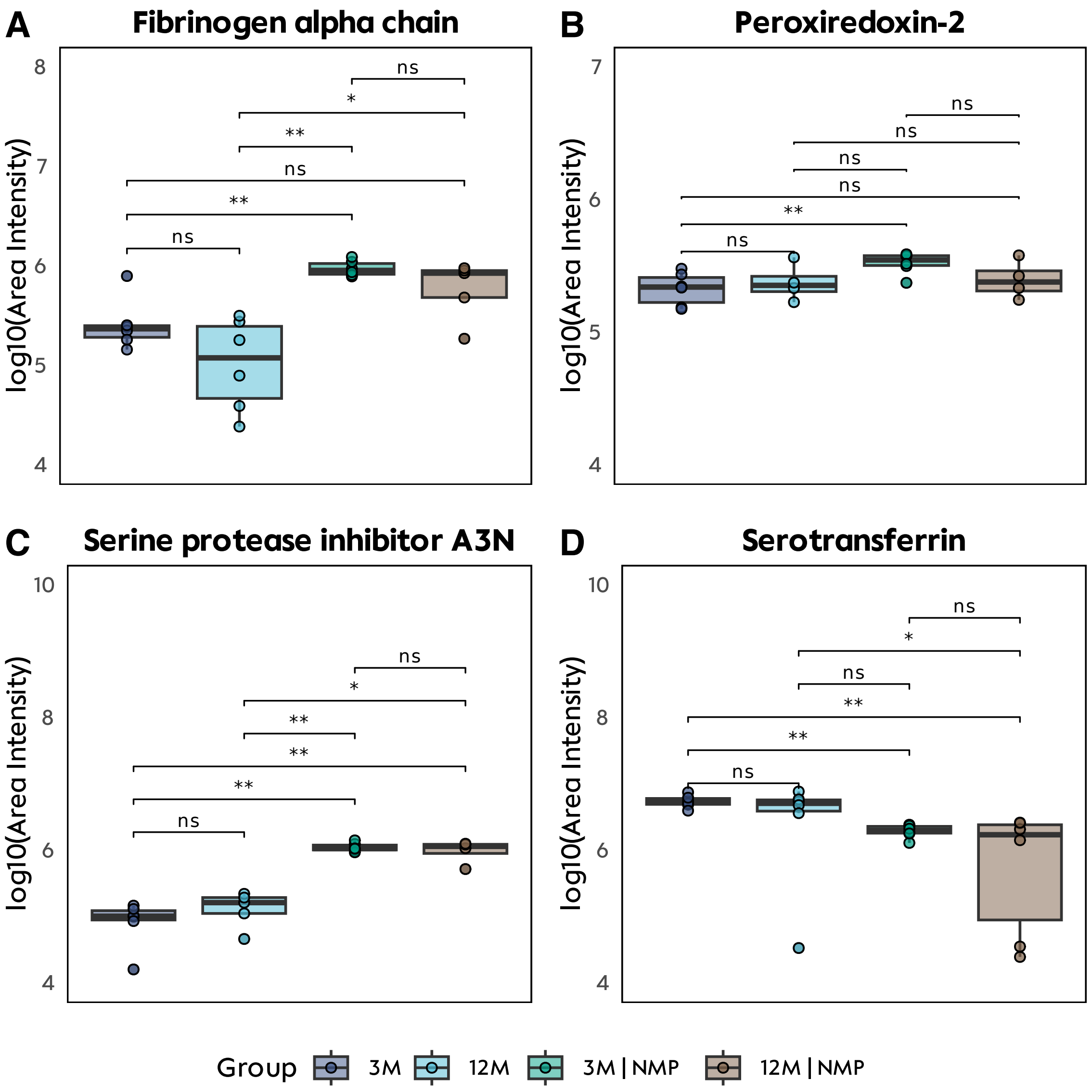

Exemplary proteins analyzed after gene ontology enrichment analysis for inflammatory response.

Supplementary Figure 4

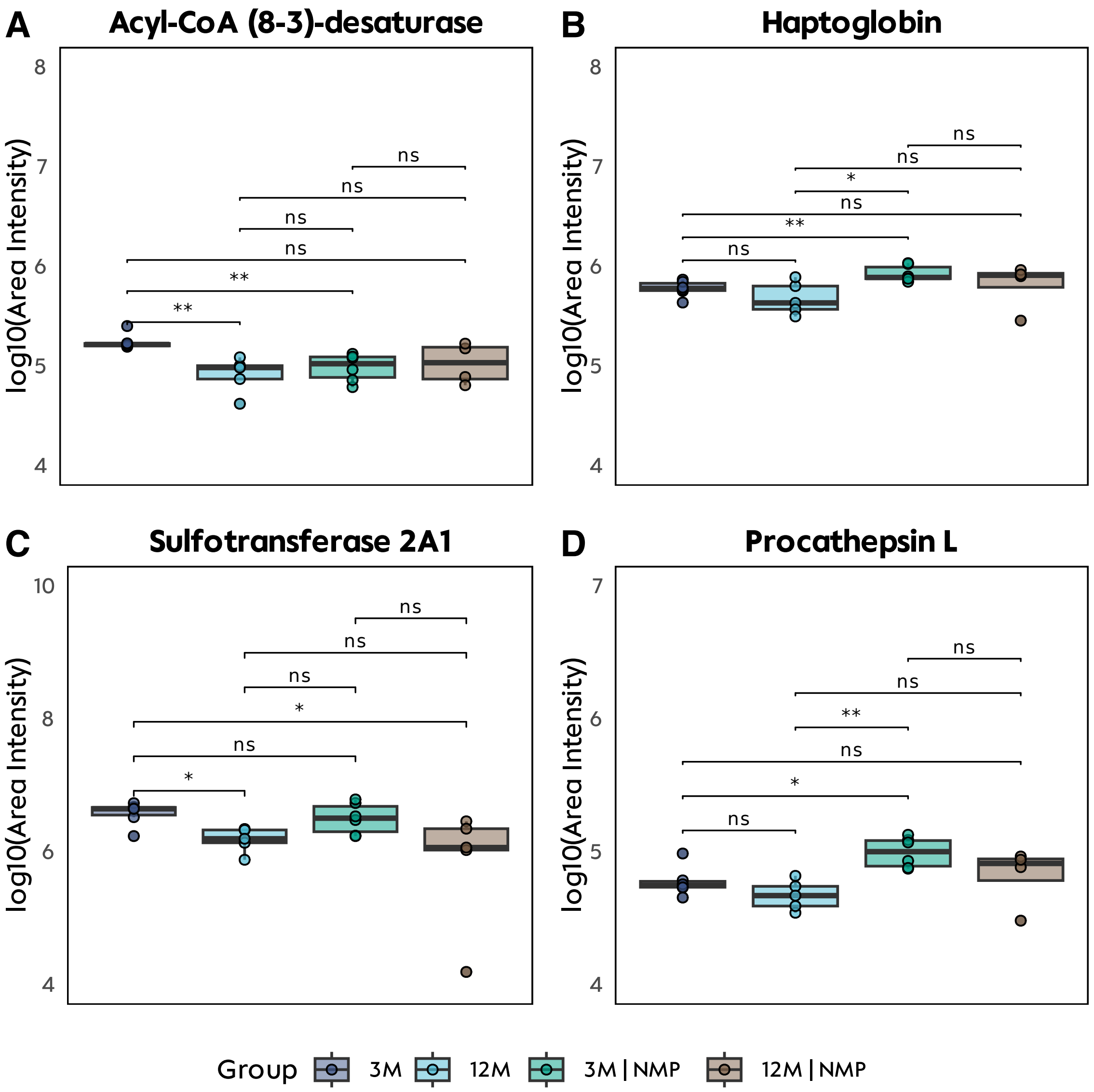

Exemplary proteins analyzed after gene ontology enrichment analysis for aging & cellular senescence.

Supplementary Figure 5

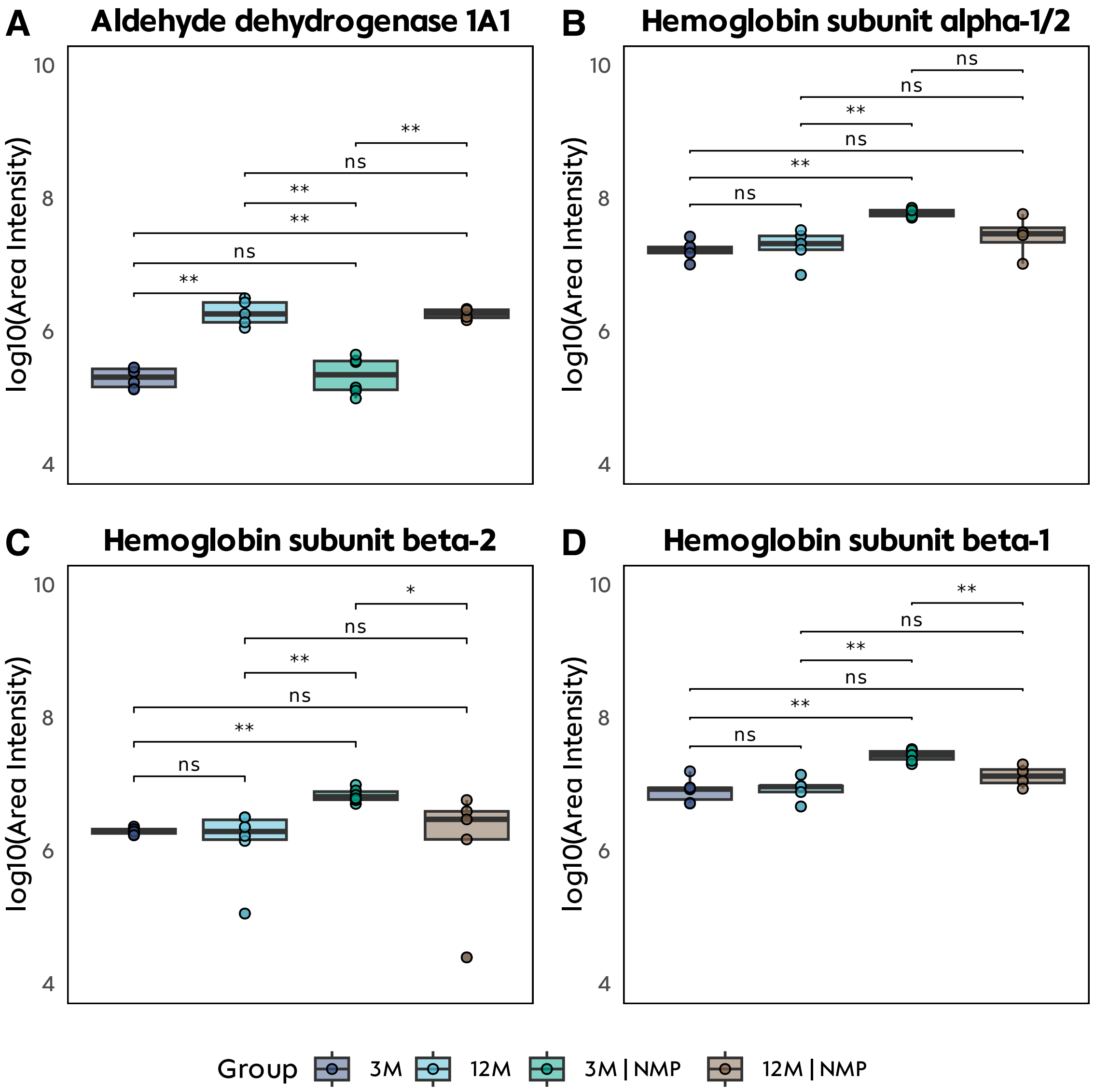

Exemplary proteins analyzed after gene ontology enrichment analysis for cellular detoxification.

Supplementary Figure 6

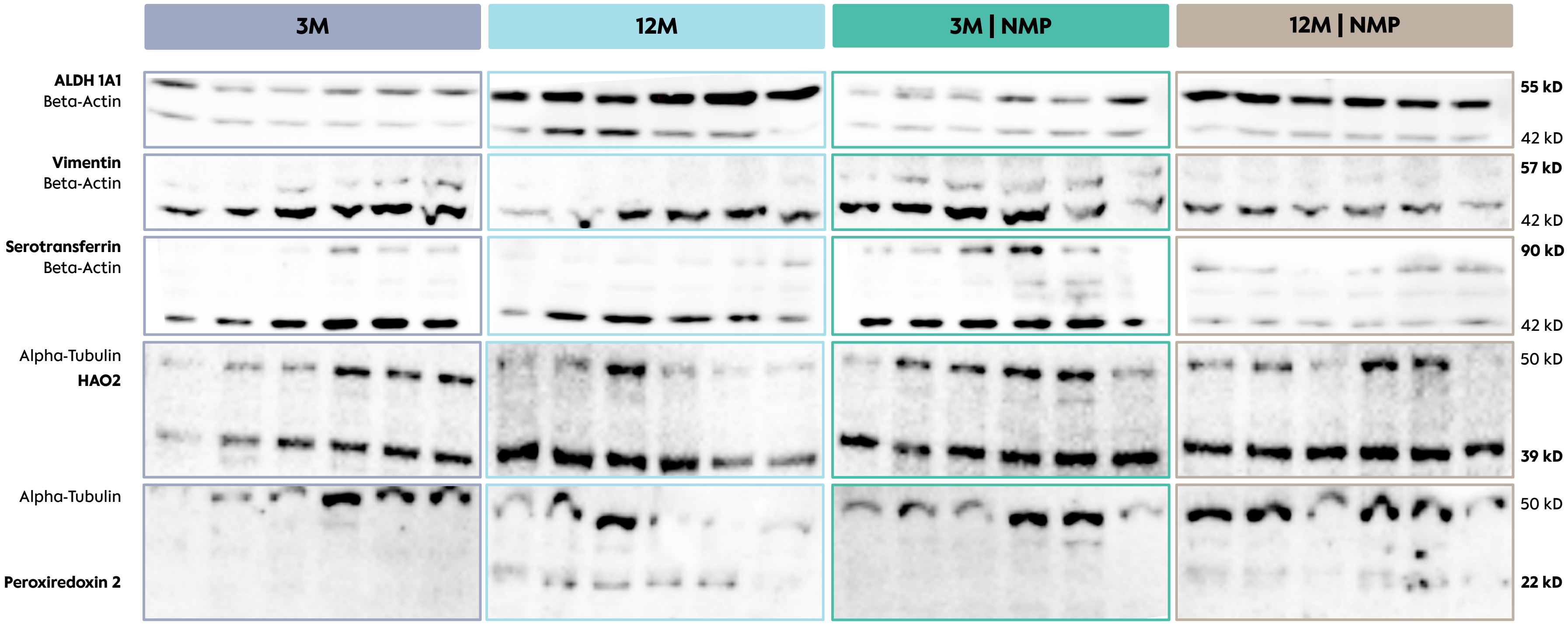

Western blot results to verify proteome analysis. Housekeeping proteins Alpha-Tubulin and Beta-Actin first two rows. From left to right: 3-month-old livers, 12-month-old livers, 3-month-old livers after NMP, and a 12-month-old livers after NMP

Supplementary Table 1

Composition of the perfusate, dialysate and heparin used in the small animal perfusion system.

**Composition of the perfusate**	**Concentration**	**Volume**	**Manufacturer**
DMEM (+)1g/L Glucose, (+) Pyruvat, (-) L-Glutamine, (-) Phenol Red)		33.85ml	Life Technologies GmbH; 11880-028
L-Alanine, L-Glutamine	200 mM	338µl	Bio&SELL GmbH; BS.K0302.0100
Penicillin/Streptomycin	10.000 U/ml	338µl	Life Technologies GmbH; 15140-122
Glucagon	1 mg/ml	0.47µl	Novo Nordisk Pharma GmbH; GlucaGen®
Insulin	100 IE/ml	4.1µl	Lilly Deutschland GmbH; EU/1/96/007/002
Heparin	5000 IE/ml	6.7µl	ratiopharm GmbH; Heparin-Natrium-25000-ratiopharm®
Dexamethason	4 mg/ml	1.4µl	Merck Healthcare Germany GmbH; 00081961
Hepes Puffer	1 M	508µl	Bio&SELL GmbH; BS.L1613
**Modified DMEM (MDMEM)**		35.05ml	
Rat erythrocytes		10ml	
Rat plasma		5ml	
		**50ml**	
Heparin	5000 IE/ml	0,2ml	
Glycine	12mM	0,5 ml	Sigma-Aldrich®
**Composition of the dialysate**			
Ci-Ca® dialysate K2		500ml	Fresenius Medical Care Deutschland GmbH
Glycine	12mM	5ml	
		505ml	
**Heparin perfusor**			
Saline solution		9ml	
Heparin	5000 IE/ml	1ml	
Glycine	12mM	500µl	
Syringe driver flow		0.6ml/h	
		300IE/h	

Supplementary Table 2

Tabular data of experiements.

		Control		Normothermic Perfusion		
**Variable**	**Overall** N = 24[1]	**3M** N = 6[1]	**12M** N = 6[1]	**3M | NMP** N = 6[1]	**12M | NMP** N = 6[1]	**p-value**[2]
**Animal weight (g)**	622 (532, 779)	485 (436, 546)	810 (754, 861)	538 (526, 551)	749 (706, 793)	<0.001
**Liver weight (g)**	18.3 (16.9, 20.2)	16.3 (15.1, 16.8)	21.5 (19.5, 24.6)	17.2 (17.0, 17.4)	20.2 (19.0, 20.2)	0.001
**Cold ischemia time (min)**	46 (41, 51)			52 (50, 54)	41 (38, 43)	0.004
**Arterial pressure (mmHg)**						
T0	45 (33, 50)			51 (46, 66)	36 (33, 43)	0.078
T1	37 (32, 42)			42 (37, 47)	33 (28, 39)	0.11
T2	41 (32, 45)			45 (43, 45)	32 (29, 38)	0.055
T3	42 (32, 73)			76 (49, 91)	31 (28, 40)	0.016
T4	44 (34, 56)			55 (46, 57)	32 (28, 43)	0.11
T5	40 (34, 54)			44 (39, 50)	35 (29, 54)	0.4
T6	34 (22, 41)			37 (8, 41)	31 (25, 39)	>0.9
**Portal vein pressure (mmHg)**						
T0	7.05 (4.95, 7.65)			4.90 (4.28, 6.20)	7.55 (7.50, 7.75)	0.054
T1	6.30 (4.48, 7.23)			4.70 (4.43, 5.58)	7.00 (6.85, 7.68)	0.2
T2	6.35 (5.00, 8.10)			5.40 (5.00, 6.18)	7.55 (6.63, 9.75)	0.15
T3	6.65 (5.45, 7.40)			5.70 (5.35, 6.58)	7.50 (6.70, 9.05)	0.11
T4	6.45 (5.55, 7.20)			5.65 (5.45, 6.15)	7.20 (6.60, 7.95)	0.078
T5	5.70 (5.50, 7.38)			5.60 (5.30, 5.60)	7.45 (6.18, 8.80)	0.076
T6	5.70 (4.90, 6.00)			5.55 (4.90, 5.75)	7.85 (5.40, 9.88)	0.2
**Bile (mg)**						
T1	458 (400, 518)			475 (424, 545)	456 (346, 495)	0.6
T2	758 (676, 848)			805 (709, 869)	735 (480, 790)	0.4
T3	749 (643, 879)			716 (621, 846)	749 (715, 1,268)	0.6
T4	656 (624, 1,175)			899 (646, 1,443)	656 (580, 955)	0.6
T5	616 (557, 1,023)			810 (453, 1,162)	616 (583, 775)	0.9
T6	614 (529, 757)			553 (404, 688)	680 (603, 808)	0.3
**Bile pH**						
T1	7.87 (7.73, 8.13)			7.95 (7.77, 8.18)	7.87 (7.66, 7.92)	0.6
T2	8.05 (7.79, 8.18)			8.18 (7.99, 8.22)	7.75 (7.69, 8.05)	0.044
T3	8.08 (7.99, 8.15)			8.12 (8.10, 8.26)	7.97 (7.90, 8.04)	0.045
T4	7.94 (7.67, 8.05)			7.73 (7.54, 7.93)	8.00 (7.93, 8.07)	0.2
T5	7.90 (7.83, 8.03)			7.86 (7.82, 7.90)	8.02 (7.89, 8.06)	0.3
T6	8.17 (8.16, 8.28)			8.21 (8.17, 8.28)	8.16 (8.03, 8.24)	0.2
**ALT (U/l)**						
T0	52 (44, 70)			47 (39, 54)	61 (48, 77)	0.2
T3	175 (133, 204)			164 (132, 195)	175 (173, 217)	0.7
T6	226 (199, 308)			201 (174, 233)	272 (213, 592)	0.15
**AST (U/l)**						
T0	63 (51, 84)			67 (59, 84)	56 (47, 79)	0.4

		Control		Normothermic Perfusion		
**Variable**	**Overall** N = 24[1]	**3M** N = 6[1]	**12M** N = 6[1]	**3M | NMP** N = 6[1]	**12M | NMP** N = 6[1]	**p-value**[2]
T3	271 (210, 335)			275 (217, 367)	271 (224, 307)	>0.9
T6	401 (351, 557)			452 (342, 527)	397 (363, 666)	0.9
**Urea (U/l)**						
T0	11.00 (10.75, 13.00)			12.00 (11.00, 13.00)	11.00 (10.25, 12.50)	0.6
T3	34 (31, 38)			30 (29, 33)	40 (35, 49)	0.010
T6	37.5 (35.5, 43.3)			35.5 (32.5, 37.0)	45.5 (38.8, 50.0)	0.029
**Bilirubin (mg/dl)**						
T0	0.15 (0.15, 0.15)			0.15 (0.15, 0.15)	0.15 (0.15, 0.15)	
T3	0.15 (0.15, 0.15)			0.15 (0.15, 0.15)	0.15 (0.15, 0.1500)	
T6	0.15 (0.15, 0.15)			0.15 (0.15, 0.15)	0.15 (0.15, 0.15)	0.3
**LDH (mg/dl)**						
T0	582 (449, 690)			550 (436, 673)	582 (571, 691)	0.6
T3	2,956 (2,353, 3,661)			3,371 (2,514, 3,779)	2,799 (2,421, 2,956)	0.9
T6	5,278 (4,415, 5,756)			5,493 (4,836, 5,781)	4,899 (4,140, 5,675)	0.7
**Bile LDH (mU/ml)**						
T1	4 (2, 8)			4 (2, 7)	5 (1, 15)	0.5
T3	5 (3, 14)			4 (3, 5)	7 (4, 32)	0.15
T6	107 (75, 275)			69 (52, 89)	295 (197, 370)	0.009
**Lactate arterial (mg/dl)**						
T0	60 (51, 66)			57 (53, 64)	63 (53, 66)	0.6
T3	20.0 (16.8, 24.0)			23.0 (18.3, 24.8)	19.0 (16.0, 20.5)	0.2
T6	17.0 (16.5, 18.8)			17.0 (17.0, 23.8)	17.5 (14.0, 18.0)	0.6
**Lactate venous (mg/dl)**						
T0	57 (46, 64)			52 (48, 59)	64 (48, 65)	0.5
T3	18.5 (14.8, 20.8)			18.0 (15.5, 22.0)	18.5 (14.3, 19.8)	0.7
T6	14.5 (13.0, 17.8)			14.5 (14.0, 22.5)	14.5 (12.3, 16.8)	0.4
**pH**						
T0	7.45 (7.32, 7.49)			7.49 (7.47, 7.50)	7.30 (7.24, 7.42)	0.025
T3	7.17 (7.14, 7.22)			7.20 (7.19, 7.23)	7.14 (7.08, 7.15)	0.025
T6	7.10 (7.07, 7.15)			7.13 (7.09, 7.15)	7.09 (7.06, 7.12)	0.13
**Sodium (mmol/l)**						
T0	143.00 (141.75, 145.25)			142.00 (140.50, 142.75)	145.50 (144.25, 146.75)	0.024
T3	139.00 (137.00, 139.50)			137.00 (136.25, 138.50)	140.00 (139.00, 142.50)	0.012
T6	139.00 (138.00, 140.25)			138.50 (138.00, 139.00)	140.00 (139.25, 141.50)	0.10
**Potassium (mmol/l)**						
T0	5.80 (5.55, 6.23)			5.70 (5.63, 6.08)	5.90 (5.15, 6.20)	0.8
T3	3.80 (3.68, 4.23)			3.70 (3.63, 3.78)	4.25 (3.90, 4.68)	0.092
T6	4.25 (4.08, 4.75)			4.20 (4.13, 4.28)	4.80 (4.18, 4.98)	0.3
**Calcium (mmol/l)**						
T0	1.13 (1.05, 1.18)			1.13 (1.06, 1.16)	1.17 (1.04, 1.23)	0.6

		Control		Normothermic Perfusion		
**Variable**	**Overall**<br>N = 24[1]	**3M**<br>N = 6[1]	**12M**<br>N = 6[1]	**3M | NMP**<br>N = 6[1]	**12M | NMP**<br>N = 6[1]	**p-value**[2]
T3	0.40 (0.36, 0.47)			0.36 (0.34, 0.42)	0.44 (0.39, 0.54)	0.078
T6	0.30 (0.28, 0.35)			0.29 (0.28, 0.31)	0.34 (0.30, 0.41)	0.2
**Chloride (mmol/l)**						
T0	119.00 (117.75, 121.00)			119.50 (119.00, 120.75)	118.00 (117.25, 120.25)	0.4
T3	113.0 (113.0, 113.3)			113.0 (113.0, 113.0)	113.0 (112.3, 113.8)	0.6
T6	112.50 (111.00, 114.25)			111.00 (111.00, 111.75)	114.50 (114.00, 115.00)	0.007
**Glucose (mg/dl)**						
T0	376 (337, 390)			355 (335, 378)	387 (349, 470)	0.2
T3	212 (203, 242)			206 (199, 213)	228 (211, 269)	0.13
T6	201 (192, 220)			201 (195, 208)	208 (188, 233)	0.5
**Bicarbonate (mmol/l)**						
T0	13.65 (12.85, 14.40)			13.50 (12.98, 14.10)	13.75 (12.90, 14.75)	0.9
T3	11.40 (10.45, 11.93)			11.75 (11.30, 11.98)	10.65 (9.93, 11.53)	0.2
T6	8.60 (7.60, 8.98)			8.80 (8.63, 9.13)	8.00 (7.45, 8.55)	0.15
**Haemoglobin (mg/dl)**						
T0	4.80 (4.45, 5.30)			5.00 (4.45, 5.40)	4.80 (4.60, 4.80)	0.4
T3	4.80 (4.60, 5.20)			4.95 (4.65, 5.18)	4.70 (4.50, 5.05)	0.8
T6	4.60 (4.20, 4.90)			4.65 (4.28, 4.88)	4.60 (4.20, 4.75)	0.9
**Haematocrit (%)**						
T0	15.30 (14.20, 16.70)			15.85 (14.23, 17.03)	15.20 (14.60, 15.30)	0.4
T3	15.40 (14.60, 16.40)			15.75 (14.80, 16.33)	15.10 (14.45, 16.10)	0.8
T6	14.70 (13.50, 15.70)			14.85 (13.73, 15.60)	14.70 (13.50, 15.20)	0.9
**pO2 arterial (mmHg)**						
T0	461 (453, 495)			460 (451, 485)	478 (456, 499)	0.4
T3	437 (424, 447)			435 (426, 439)	442 (427, 460)	0.5
T6	448 (433, 468)			455 (428, 470)	448 (437, 457)	0.9
**pO2 venous (mmHg)**						
T0	39 (36, 43)			38 (35, 42)	39 (38, 43)	0.4
T3	42.7 (39.2, 43.9)			43.6 (40.2, 44.0)	41.2 (39.5, 43.1)	0.6
T6	38.1 (33.7, 40.6)			38.4 (34.8, 41.4)	37.0 (32.9, 39.9)	0.7

[1] Median (IQR), [2] Kruskal-Wallis rank sum test. Raw data gathered during liver machine perfusion.

SUPPLEMENTARY METHODS

Perfusion Setup and Procedure

We used a modified version of a small animal NMP system previously published by our group.(1) Priming volume of the entire perfusion system was 50 ml. The perfusion circuit includes a custom-designed perfusion chamber that enables the liver to be stored in its own perfusate while floating freely. Two circuits emerge from this chamber. A perfusion circuit that provides oxygenation of the blood and a perfusion circuit dialysis unit. For this purpose, a silicon membrane oxygenator (*Radnoti LLC., Dublin, Ireland*) with a capacity of 10ml and 90% O2 atmosphere was connected downstream. A glass bubble trap protects the liver graft from air embolisms. To facilitate dual-vessel perfusion, two pumps were employed. The first pump, set to a rate of 1.2 ml/min/g liver weight, generated the overall flow. A portal flow rate of 1 ml/min/g liver weight was maintained, while a second roller pump diverted 0.2 ml/min/g liver weight from the portal circuit for arterial perfusion. Circuit pressures were continuously measured and recorded with BDAS 2.0 software (*Harvard Apparatus, Holliston, MA*). To regulate arterial pressure, we implemented a cutoff of 110 mmHg, after which a 100 mg metamizole bolus was administered, following previously established protocols. (1)

The second circuit was used for dialysis. In the countercurrent principle, the perfusate flows against the dialysate. Flow was set to 10 ml/min for both sides. After passing through the dialysis, the perfusate was collected in the perfusion chamber and provided buoyancy to the liver. Heparin was continuously infused with 300 IE/h using a syringe driver. Three-way stopcocks behind the oxygenator, chamber and dialysis allowed for perfusate sampling.

Composition of the perfusate and dialysate

Each experiment required 50 ml of perfusion medium. As basic stock we used 35ml of modified Dulbecco's Modified eagle Medium (DMEM) which was supplemented with L-Alanine/L-Glutamine, Penicillin/Streptomycin, Glucagon, Insulin, Heparin, Fortecortin and Hepes Buffer. As an oxygen carrier we used 10 ml of rat erythrocytes, which were harvested

for each experiment. Additionally, we added 5 ml of rat plasma. Before perfusion start, we supplemented 1000IE Heparin and 12 mM glycine (**Supplementary Table 1**). We used 500 ml of Ci-Ca® dialysate K2 (*Fresenius Medical Care, Bad Homburg, Germany*) with the addition of 5ml glycine for same concentration as in perfusate as a dialysate.

Perioperative and Surgical procedures

Before surgery, medication for anesthesia as well as cold Histidine-Tryptophan-Ketoglutarate (HTK) solution were prepared. We utilized a 4°C HTK solution, for flushing. Anesthesia was inducted with inhaled isoflurane and followed by subcutaneous application of buprenorphine and ketamine. The rat was fixed, and the abdomen was disinfected. After checking for a pain stimulus, the abdominal cavity was opened. The liver was mobilized from its ligaments. Using a customized cannula, the bile duct was cannulated and checked for adequate drainage. The hepatic arteria was prepared down to the Aorta and vascular outlets were ligated. 500IE of heparin in 1 ml Ringer solution was injected into the abdominal vena cava inferior. The abdominal aorta was cannulated for blood collection and flush. Collected blood was treated as previously described.(2) The thoracal cavity was opened, and the thoracic aorta was clamped. 20 mL of prepared HTK solution were given throw the Aorta to flush the liver. Additionally, the portal vein was cannulated and flushed with 20mL of HTK. Time between blood collection and cold flush (warm ischemia time, WIT) did not exceed 5 minutes. The artery was then cannulated using an aortic patch. The esophageal vein was ligated, and the Vena cava inferior was opened near the liver. Carefully, the liver was mobilized from the rest of its ligaments and preserved into a pre-weighed container filled with the same cold HTK solution as described above. The liver was weighed before perfusion start. Before implanting the liver in the perfusion system and after the end of the experiment, the liver was flushed again with saline solution.

Quantification of liver proteins.

The search was performed in PEAKS Studio (*Bioinformatics Solutions Inc., Waterloo, Canada*) with the following set of (i) parent mass error tolerance 20.0 ppm, (ii) fragment mass error tolerance 0.05 Da, (iii) precursor mass search type monoisotopic, (iv) enzyme trypsin, (v) max missed cleavages 3, (vi) variable modifications: Oxidation (M), deamidation (NQ) and acetylation (N-term), max variable PTM per peptide 3. taxonomy Swiss-Prot database of rattus norvegicus. A peptide significance filter -lgP >20, a protein significance filter -lgP >15 and unique-peptide filter was set to 1. Label-free quantification with PEAKS Q was used and allowed to autodetect the reference sample and align the sample runs. The protein significance filter was set to 0 unique peptide filter to 1 and protein fold change filter to 1 in the export settings.

After adjusting for multiple testing using the method proposed by Benjamini & Hochberg a q-Value <0.05 was used as a cut-off to create a heatmap of the top proteins with the largest intergroup difference. Gene ontology (GO) enrichment analysis were performed using the rat genome database of the Multi Ontology Enrichment Tool (MOET) version 2.(5) Pathways of particular interest were cellular senescence, inflammatory response, and cellular detoxification. Proteins of interest were cross-referenced with available literature and then selected for validation with western blotting.

Western blot analysis

Approximately 100 mg of liver tissue was homogenized with the help of a mixer mill (*MM400, Retsch GmbH, 42781 Haan, Germany*) and solubilized with a RIPA buffer (1% NP-40, 0.5% SDS, 0.1% sodium deoxycholate, 0.01% PMSF, and PBS). Next, samples were incubated on ice for 5 minutes and centrifuged at 12,000g at 4°C for 45 minutes. BCA (*Sigma-Aldrich, St. Louis, MO 63178, USA*) was used to determine protein load after 1:100 dilution of samples and measured by a FLUOstar OPTIMA Microplate Reader (*BMG LABTECH GmbH, 77799 Ortenberg, Germany*). 5-15% acrylamide gels with 15 µg protein load per well were used for

SDS-PAGE. Proteins were blotted using the iBlot2 transfer stacks with PVDF membranes (*Thermo Fischer Scientific, Waltham, MA, USA*). We used recombinant Anti-vimentin antibody (ab8069, diluted 1:1,000), recombinant Anti-transferrin receptor antibody (ab269513, diluted 1:5000), Anti-HAO2 antibody (ab229817, diluted 1:1000), Anti-peroxiredoxin 2 antibody (ab226922, diluted 1:2500) Anti-aldehyde dehydrogenase 1A1 antibody (ab9883, diluted 1:2000) (all *Abcam, Cambridge, UK*) as primary antibodies. Blots were detected using the Pierce ECL Western (*Thermo Fischer Scientific, Waltham, MA, USA*) with horseradish peroxidase-conjugated secondary anti-bodies Goat Anti-Mouse IgG H&L (ab205719, diluted 1:10,000), Goat Anti-Rabbit IgG H&L (ab205718, diluted 1:20,000), and Donkey Anti-Goat IgG H&L (ab205723, diluted 1:20,000) (all Abcam, Cambridge, UK) using the *Bio-Rad ChemiDoc XBS Quantity One 4.6.9* (*Bio-Rad Laboratories GmbH, 85622 Feldkirchen, Germany*). Housekeeping proteins were measured with Anti-actin antibody (A2103, diluted 1:8,000) and Anti-alpha-tubulin antibody (T9026, diluted 1:500) (both *Sigma-Aldrich, St. Louis, USA*).

References for Supplementary Methods:

1. Claussen F, Gassner J, Moosburner S, Wyrwal D, Nosser M, Tang P, et al. Dual versus single vessel normothermic ex vivo perfusion of rat liver grafts using metamizole for vasodilatation. PLoS One. 2020;15(7):e0235635.
2. Gassner J, Nosser M, Moosburner S, Horner R, Tang P, Wegener L, et al. Improvement of Normothermic Ex Vivo Machine Perfusion of Rat Liver Grafts by Dialysis and Kupffer Cell Inhibition With Glycine. Liver Transpl. 2019;25(2):275-87.
3. Ciordia S, Alvarez-Sola G, Rullan M, Urman JM, Avila MA, Corrales FJ. Digging deeper into bile proteome. J Proteomics. 2021;230:103984.
4. Wiśniewski JR, Zougman A, Nagaraj N, Mann M. Universal sample preparation method for proteome analysis. Nature Methods. 2009;6(5):359-62.
5. Vedi M, Nalabolu HS, Lin C-W, Hoffman MJ, Smith JR, Brodie K, et al. MOET: a web-based gene set enrichment tool at the Rat Genome Database for multiontology and multispecies analyses. Genetics. 2022;220(4).
